# The autophagy switch: A critical determinant of arsenic-induced carcinogenesis and cancer therapy

**DOI:** 10.1016/j.toxrep.2026.102229

**Published:** 2026-02-22

**Authors:** Marzieh Zeinvand-Lorestani, Fakher Rahim, Hamed Zeinvand-Lorestani

**Affiliations:** a Department of Chemical Technologies, Iranian Research Organization for Science and Technology (IROST), Tehran, Iran; b Faculty of Medicine, Department of Medical Biology, Kocaeli University, Kocaeli 41001, Türkiye; c Department of Internal Medicine, Osh State University, Osh City, Kyrgyzstan; d Department of Toxicology, Faculty of Pharmacy, Ahvaz Jundishapur University of Medical Sciences, Ahvaz, Iran

**Keywords:** Autophagy, Autophagy switch, Arsenic exposure, Carcinogenesis, Oxidative stress

## Abstract

Arsenic, a widespread environmental toxicant and unexpectedly effective chemotherapeutic agent, has complex and significant effects on cellular homeostasis. Autophagy, a conserved lysosomal degradation process, plays a key role in arsenic's dual functions as a carcinogen and a treatment. While current reviews have documented interactions between arsenic and autophagy, this review introduces a new conceptual model: the “Autophagy Switch.” We propose that the cellular choice between autophagy-assisted survival and autophagy-dependent death is not simply black and white but exists within a dynamic balance called the Arsenic Contextual Triad—comprising chemical form, exposure pattern (dose and duration), and the cell’s oncogenic background. We compile evidence showing how this switch influences outcomes across the cancer spectrum, from promoting skin cancer through p62/Nrf2 feedback loops to breaking down oncogenic factors like PML-RARα and BCR-ABL in leukemia. Additionally, we critically assess the therapeutic potential of targeting this switch, emphasizing how drugs that either inhibit or promote autophagy can work together with arsenic trioxide (ATO) to combat drug resistance in solid tumors such as glioblastoma and ovarian cancer. By shifting from simple descriptions to a detailed mechanistic and contextual understanding, this review offers a valuable guide for future research aiming to harness the autophagy switch for cancer prevention and personalized treatment.

## Highlights

• Arsenic exposure activates autophagy, influencing cancer initiation and progression.
• Autophagy functions as a dual switch, either promoting tumor survival or inducing cell death.
• Understanding the autophagy switch is critical for arsenic-related cancer risk assessment.
• Modulating autophagy offers promising strategies for targeted cancer therapy.

## Graphical Abstract

**Figure:**
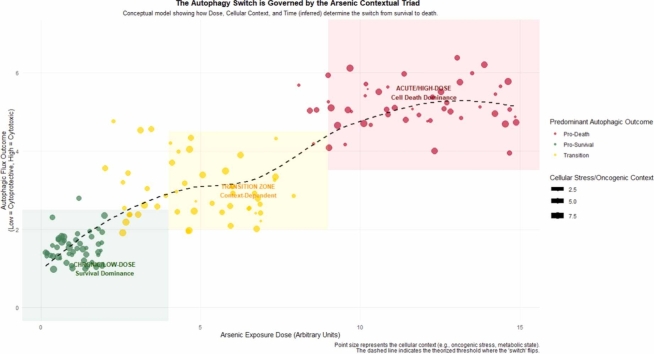
The Arsenic Contextual Triad and Autophagy Switch.This figure creates a conceptual, data-inspired plot showing how the three factors of the “Arsenic Contextual Triad” influence the autophagic outcome. The color of the points indicates whether the outcome is primarily pro-survival (green), transitional/mixed (yellow), or pro-death (red). The size of the points represents the third variable in the triad: Cellular Stress/Oncogenic Context. A smoothed trend line shows the theoretical transition. Annotated zones clearly label the conceptual spaces where the “Autophagy Switch” favors survival or death. This figure visually encapsulates the core novel thesis of the manuscript.

## 1 Introduction

Arsenic is a widespread environmental and industrial contaminant, frequently detected in groundwater and food sources worldwide. Human exposure predominantly occurs through ingestion of contaminated drinking water derived from natural geological sources. Populations in countries such as Bangladesh, India, Iran, China, Argentina, and Mexico are particularly affected, consuming water with elevated arsenic concentrations. Chronic exposure has been linked to a range of pathological outcomes, including dermatological abnormalities, cardiovascular disorders, hepatic injury, neurological dysfunction, and cancer [1], [2], [3].

Paradoxically, arsenic trioxide (ATO) has been recognized as a breakthrough treatment for acute promyelocytic leukemia (APL), achieving remarkable remission rates by targeting the PML-RARα oncoprotein. Clinical studies have demonstrated that ATO induces high complete remission rates and durable responses in both relapsed and newly diagnosed patients. Evidence from prospective trials and real-world studies shows that ATO-based regimens significantly improve event-free and overall survival compared with conventional therapies. In addition, meta-analyses and long-term follow-up studies confirm enhanced molecular remission, disease-free survival, and overall treatment outcomes in APL patients receiving ATO [4], [5], [6], [7], [8], [9]. Collectively, these findings support the characterization of ATO as a transformative therapy that has shifted APL from a highly fatal disease to one of the most curable forms of leukemia.

The mechanisms behind this duality are mainly linked to the induction of oxidative stress and apoptosis. Studies have shown that arsenic and its metabolites can induce oxidative DNA damage, alter DNA methylation patterns, promote genomic instability, and affect DNA repair, cell proliferation, and programmed cell death pathways. Lipid peroxidation, driven by reactive oxygen species (ROS), is recognized as a central mechanism in arsenic-mediated cellular injury and genotoxicity across both in vitro and in vivo models [10].

Emerging evidence indicates that arsenic toxicity is not limited to oxidative stress mechanisms. It is increasingly evident that another cellular process—autophagy—also plays a similarly essential and complex role. Autophagy, a complex intracellular recycling system, maintains cellular health by clearing damaged organelles and aggregated proteins. In cancer, its role is highly context-dependent, acting as a tumor suppressor by preventing genomic instability and as a tumor promoter by enhancing metabolic fitness under stress. Unlike apoptosis, autophagy may function protectively or contribute to cytotoxicity, depending on cell type and stress intensity. Numerous studies have explored arsenic-induced modulation of autophagy pathways, highlighting its dual role as both a toxicant and a potential therapeutic agent [11], [12].

Previous reviews on arsenic and autophagy have effectively summarized data from various studies. However, they often lack a unifying explanation for the contradictory outcomes—why autophagy sometimes protects cells from arsenic toxicity and other times causes cell death. We addressed that critical gap: arsenic does not simply induce or inhibit autophagy, but rather toggles a molecular switch that can be manipulated for therapeutic benefit. The present study aims to introduce and define the Arsenic Contextual Triad that controls the autophagy switch, summarize evidence across cancer types to show how this switch functions in both carcinogenesis and treatment, and critically evaluate the potential for targeting this switch, discussing current challenges and future directions for using autophagy modulation in arsenic-related diseases.

## 2 KEGG-style overview: macroautophagy in cancer under arsenic exposure

Macroautophagy is a highly conserved catabolic mechanism essential for maintaining cellular homeostasis by sequestering damaged organelles and aggregated proteins for lysosomal degradation. Three primary autophagic pathways have been characterized: macroautophagy (the predominant form), microautophagy, and chaperone-mediated autophagy. Macroautophagy is orchestrated by autophagy-related (ATG) proteins, which regulate key steps including membrane nucleation, phagophore expansion, and autophagosome maturation. According to KEGG pathway annotations, the process can be divided into initiation, nucleation, elongation/closure, and lysosomal fusion. Initiation is controlled by the ULK1 kinase complex, followed by vesicle nucleation mediated by the Beclin-1/class III PI3K complex, with microtubule-associated protein light chain 3 (LC3) playing a critical role during membrane elongation and autophagosome formation [13], [14], [15].

**Fig. 1:**
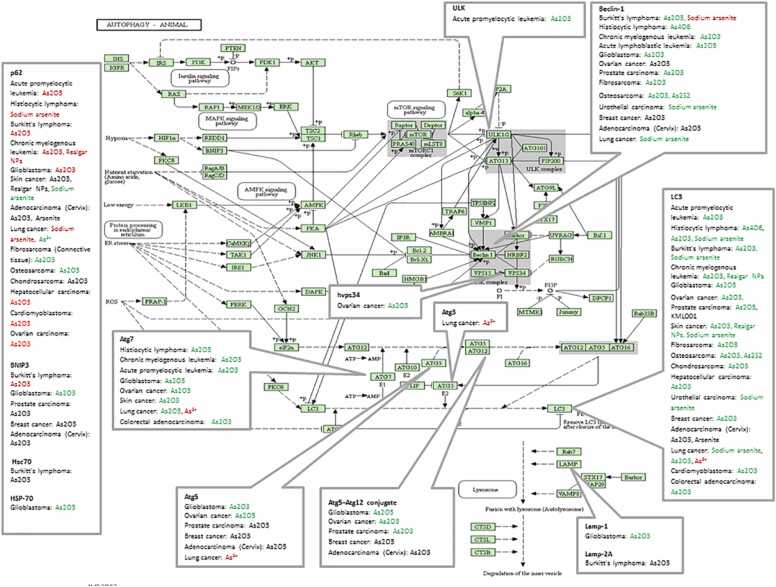
KEGG-style overview: Autophagy in cancer under arsenic exposure. Up regulated genes are indicated by green and the down regulated genes are indicated by red.

In cancer contexts, alterations in these canonical autophagy markers—whether through upregulation or downregulation following arsenic exposure—indicate that autophagy may significantly influence arsenic-associated cellular outcomes, including cell survival, programmed death, or therapeutic response (Fig. 1).

## 3 Integrating the role of the arsenic microbiome metabolite, DMA(III)

**Fig. 2:**
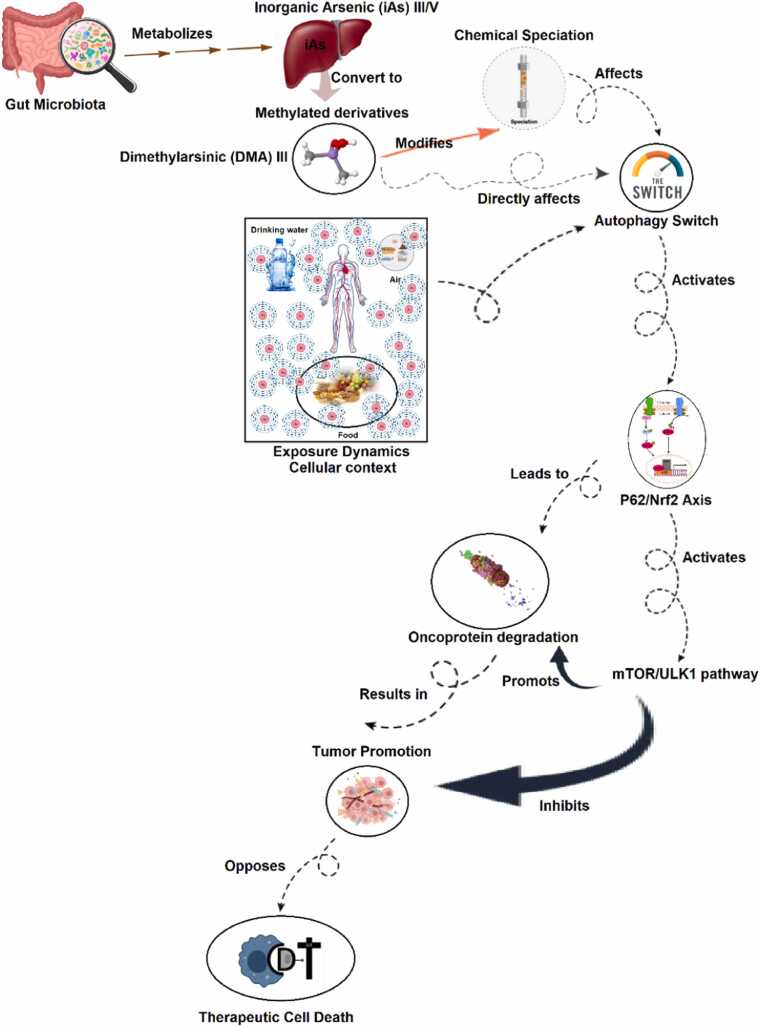
The interaction between arsenic, microbiome and autophagy.This schematic outlines a hypothesized oncogenic pathway. Environmental inorganic arsenic is metabolized by gut bacteria to DMA III, a key signaling molecule. In susceptible cells, DMA III flips an autophagic switch, initiating a P62/Nrf2 response. This response has a dual pro-tumor effect: it engages the mTOR/ULK1 pathway—which in this context suppresses protective autophagy—and enhances the degradation of specific oncoproteins, potentially altering cell survival signals. The integrated outcome is the suppression of therapeutic cell death pathways and the creation of a cellular environment conducive to tumor development and progression.

Most reviews focus on inorganic arsenic (iAs) and ATO. However, the human microbiome metabolizes arsenic into trivalent methylated species, such as dimethylarsinite (DMA(III)), which are highly toxic and genotoxic. Linking this to autophagy is an entirely novel perspective. A critical and largely overlooked aspect of arsenic biology is its biotransformation by the human microbiome. Gut microbiota convert inorganic arsenic into trivalent methylated species, most notably dimethylarsinite (DMA(III)), which displays potent toxicity and genotoxicity [16]. Recent evidence indicates that the microbiome can directly influence host autophagic responses [17]. Arsenic and its methylated metabolites, including DMA(III) and MMA(III), have been shown to induce oxidative stress and modulate key cellular pathways, including autophagy and apoptosis, in various tissues [18], [19]. Studies indicate that both direct arsenite exposure and microbiome-derived arsenic metabolites can disrupt autophagic regulation in adipose and muscle tissues, contributing to metabolic dysfunction and cellular stress [20], [21]. Trivalent methylated metabolites such as DMA(III) and MMA(III) are highly reactive and biologically active, with MMA(III) exhibiting greater toxicity and stronger inhibition of metabolic enzymes, such as pyruvate dehydrogenase, than inorganic arsenite [18], [22]. Emerging evidence points to a dynamic interplay between autophagic flux and the p62–NRF2 signaling axis as a key determinant of cellular responses to arsenic exposure. Disruption of autophagy can lead to p62 accumulation and sustained NRF2 activation, reshaping oxidative-stress signaling and influencing cell fate in a context-dependent manner. In parallel, trivalent arsenic metabolites such as DMA(III) may function as an “autophagy switch” in certain cell types by engaging P62/NRF2 signaling and modulating the mTOR/ULK1 pathway; although this pathway classically regulates autophagy initiation, arsenic-related stress can shift it toward impaired or dysregulated autophagic activity rather than complete inhibition. The P62/NRF2 axis may also contribute to the turnover of selected oncoproteins, yet its persistent activation can, in some tumor contexts, reduce sensitivity to therapy-induced apoptosis and support cellular adaptation and survival. Collectively, these findings suggest that environmental arsenic—potentially shaped by gut microbiota-driven biotransformation into reactive intermediates such as DMA(III)—can perturb autophagy homeostasis and cell-death signaling in a manner that is highly cell-type and exposure dependent, with outcomes ranging from stress adaptation to tumor progression [23], [24]. Evaluations on arsenic carcinogenesis highlight that methylated arsenic species retain high biological activity and may interfere with key signaling pathways, including those regulating oxidative stress and autophagy [23], [25], [19]. Moreover, arsenic exposure has been shown to disrupt autophagy regulation in metabolic tissues, supporting a possible link between arsenic metabolites and autophagy signaling. Uncovering this 'microbiome-arsenic-autophagy' axis could transform our understanding of individual susceptibility to arsenic-related diseases and the variable efficacy of ATO therapy, opening new possibilities for interventions targeting the gut microbiome (Fig. 2).

## 4 Immunomodulatory role of arsenic-induced autophagy (immunophagy)

The intersection of autophagy, arsenic, and the immune system is a nascent and high-impact field. ATO has been shown to have immunomodulatory effects, and autophagy is crucial for antigen presentation and immune cell function. Beyond its direct cytotoxic effects on cancer cells, arsenic trioxide modulates the tumor immune microenvironment (TIME). ATO can inhibit the differentiation and function of immunosuppressive myeloid-derived suppressor cells (MDSCs) [26]. Intriguingly, autophagy is a key regulator of MDSC survival and differentiation [27]. This presents a novel, unexplored mechanism: does ATO's anti-tumor immunity rely on autophagy-dependent reprogramming of the TIME. We hypothesize that ATO-induced autophagy in tumor cells enhances antigen presentation, while simultaneously triggering autophagic pathways in immune cells to dismantle immunosuppressive networks. Understanding this crosstalk could lead to powerful combinations of ATO with immune checkpoint inhibitors, leveraging immunophagic activation to overcome resistance to immunotherapy.

## 5 Introduce the concept of autophagic collapse or autosis

The term autophagic cell death is often used loosely. Introducing the specific, morphologically distinct process of autosis — a form of cell death triggered by excessive autophagy — offers a precise, mechanistic term and a new hypothesis for ATO's action in resistant cancers. While the term autophagic cell death is frequently invoked, its mechanistic definition is often unclear. We suggest that, under conditions of high, sustained autophagic flux induced by ATO, cells may undergo a specific form of cell death termed autosis. **Autosis is a Na+ /K+ -ATPase-dependent form of cell death characterized by distinctive morphological features (e.g., ballooning of the perinuclear space) and is distinct from apoptosis and necrosis** [28]. This idea shifts the therapeutic focus: instead of simply 'inducing autophagy,' the goal in resistant tumors like GBM could be to push the autophagic process beyond a point of salvage into autotic collapse. This model explains why late-stage autophagy inhibition (e.g., with chloroquine) can synergize with ATO—by blocking the completion of a prosurvival process, it traps the cell in an autophagic crisis. Future studies should examine autosis markers in ATO-treated cancer models, potentially identifying Na+ /K+ -ATPase as a new therapeutic target to enhance ATO's effectiveness.

## 6 Autophagy and disease in arsenic exposure

**Fig. 3:**
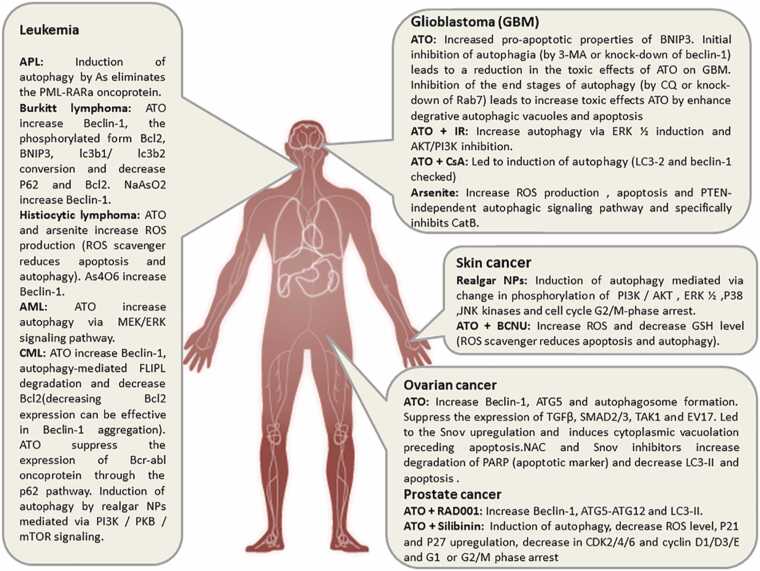
Autophagy alteration by arsenic exposure in cancerous diseases.

Alterations in autophagic flux have been observed in a wide range of pathological conditions following arsenic exposure, encompassing both malignant and non-malignant states. The impact of arsenic on autophagy is highly context-dependent, determined by factors such as exposure dose and duration, cell type, and the nature of intracellular stressors. Current evidence indicates that arsenic-induced autophagy can exert either cytoprotective or cytotoxic effects [29]. This review integrates these findings, highlighting the complex interplay between autophagy and cancer mechanisms in scenarios of environmental arsenic exposure or therapeutic administration of arsenic-based compounds (Fig. 3).

## 7 The autophagy switch in arsenic-induced carcinogenesis

**Fig. 4:**
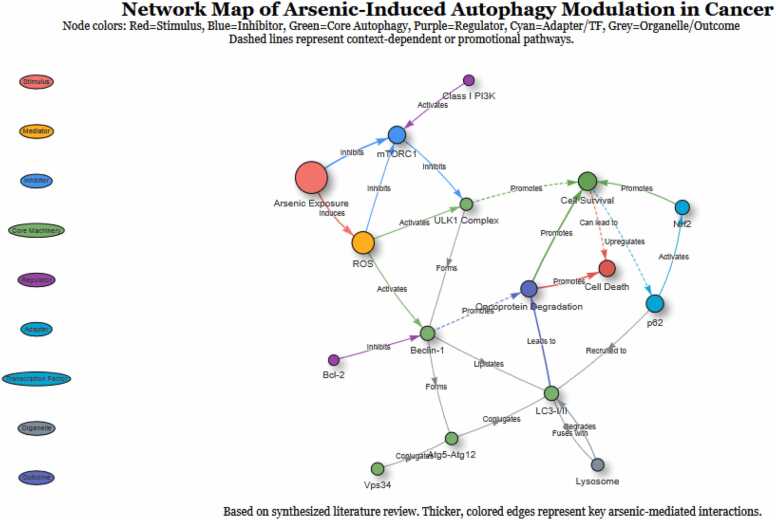
Network Perturbation of Autophagy Pathways by Arsenic.This figure creates a node-and-edge network diagram based on the KEGG pathway, visually highlighting which parts of the autophagy machinery are most significantly targeted by arsenic, based on the literature synthesized in the review. It visually demonstrates that Arsenic Exposure is a central node that influences key regulators such as ROS and mTORC1, which, in turn, affect the core autophagy machinery (ULK1, Beclin-1, LC3). Crucially, it highlights critical, arsenic-specific interactions, including Arsenic - ROS (Induces), Arsenic - mTORC1 (Inhibits), ROS - Bcl-2 (Inhibits, thereby activating Beclin-1), and p62 - Nrf2 (Activates, illustrating the pro-survival feedback loop). The divergent outcomes of Cell Survival and Cell Death are clearly shown, with context-dependent pathways represented by dashed lines.

Chronic, low-dose arsenic exposure can promote carcinogenesis, with autophagy serving as a key mediator in this process. In this context, the switch is often exploited to support cell survival and tumor initiation(Fig. 4).

### 7.1 Leukemia

The autophagic response to arsenic trioxide (ATO) has been extensively studied in acute promyelocytic leukemia (APL), particularly in NB4 cells, where ATO promotes degradation of the oncogenic PML–RARA fusion protein via autophagy, contributing to therapeutic remission. The effectiveness of ATO in treating APL is closely related to autophagy. The drug promotes the autophagic degradation of the oncogenic PML-RARα fusion protein, a process that depends on p62 and ULK1 [30], [31], [32], [33], [34].

In HL-60 cells, findings are inconsistent: some studies report no autophagic induction under certain arsenical treatments, whereas others describe **ATO-triggered cell death with high autophagic flux**. In Raji (Burkitt lymphoma) cells, ATO induces dose- and time-dependent growth inhibition, G2/M arrest, apoptosis, and enhanced autophagic vacuolization, accompanied by p62 degradation, Beclin-1 upregulation, and Bcl-2 downregulation [33], [35], [36], [37].

Reactive oxygen species (ROS) act as upstream mediators; ROS scavengers reduce both autophagosome formation and cell death in models such as U937 and BM2 cells, indicating that ROS-dependent induction of apoptosis or autophagy depends on cell type. Mechanistically, arsenite stimulates mitochondrial superoxide production and inhibits aconitase, generating H₂O₂ that triggers autophagy and, under severe stress, mitochondrial permeability transition and apoptosis [38], [39].

In chronic myelogenous leukemia (CML), ATO triggers p62-dependent degradation of another oncoprotein, BCR-ABL. Here, autophagy plays a vital role in executing the therapy’s effects. K562 and K562-derived initiating cells (K562s) exhibit dose-dependent and divergent autophagic responses to ATO, influencing self-renewal, resistance, and senescence. ATO robustly induces autophagy in both drug-sensitive and drug-resistant K562 derivatives, with decreased Bcl-2, Beclin-1 accumulation, and **autophagy-associated cell death**[40].

Additional mechanisms include ubiquitin-mediated regulation of apoptosis inhibitors: FLIP_L downregulation correlates with ATO-induced autophagy, and Cbl-b E3 ligase mediates FLIP_L degradation via the ubiquitin–proteasome system. p62/SQSTM1 facilitates autophagic degradation of BCR-ABL; inhibition of autophagy, cathepsin B, p62, or Atg7 partially rescues BCR-ABL–expressing progenitors from ATO effects [41]. Realgar (As₄S₄) nanoparticles similarly reduce K562 cell proliferation and degrade BCR-ABL via combined autophagic and apoptotic mechanisms that involve PI3K/AKT/mTOR signaling. In AML models, ATO activates the MEK/ERK cascade to induce autophagy, and genetic or pharmacologic inhibition of autophagy attenuates its anti-leukemic effects. In Molt-4 cells, ATO upregulates Beclin-1, with Bax contributing to **autophagy-associated cell death**[42] (Table 1).

**Table 1:** Summary of included studies in leukemia.

Contaminant	Study model		Cell line	Tissue	Disease	Marker	Findings	Ref
As2O3	Cell line		NB4	Peripheral blood	Acute promyelocytic leukemia	-	• Arsenic trioxide (As₂O₃) triggers both apoptotic and autophagic pathways in cells.	Ren et al. [34]
As2O3	Cell line		NB4 EVAsR1	Peripheral blood	Acute promyelocytic leukemia	LC3, P62	• Autophagy facilitates the degradation of the PML-RARA fusion protein, a process mediated by the p62 cargo receptor. • Co-treatment with ATO and bortezomib enhances autophagic induction in an additive manner.	Ganesan et al. [31]
			HS-5	Bone Marrow/Stroma				
	Mice		-	Transgenic mice				
	Bone marrow samples from APL patients							
As2O3	Cell line		NB4	Peripheral blood	Acute promyelocytic leukemia	LC3 II/LC3 I	• Concurrent administration of ATO with resveratrol or genistein elevates the LC3-II/LC3-I ratio in NB4 cells and neonatal rat ventricular myocytes, indicating augmented autophagy.	Fan et al. [30]
As2O3	Bone marrow (BM) mononuclear cells (MNC)				Acute promyelocytic leukemia	-	• Functional autophagy is essential for the full cytotoxic synergy of VTX and AZA. • PML::RARα may prime cells for enhanced cell death. • This priming occurs through both autophagy-dependent and autophagy-independent mechanisms.	Zaza et al. [43]
As2O3	Cell line		NB4	Peripheral blood	Acute promyelocytic leukemia	ULK1, p62, LC3	• The degradation of ectopically expressed PML-RARA during ATO treatment depends on ULK1 activity.	Isakson et al. [32]
As2O3,PAOIII,AsV,DMAV	Cell line		HL-60	Peripheral blood	Acute promyelocytic leukemia	MAP-LC3	• Tested compounds did not notably alter acridine orange staining or the subcellular localization of MAP-LC3.	Charoensuk et al. [35]
Realgar-Indigo naturalis Formula (RIF)	Cell line		HL60-PMLA216V-RARα cell line	-	Acute promyelocytic leukemia	PI3K, mTOR, P-mTOR, AKT, p-AKT and p62	• Tanshinone IIa, indirubin, and the total saponins from Radix Pseudostellariae were found to potentiate the effects of tetra-arsenic tetra-sulfide by downregulating PMLA216V-RARα, likely through mTOR pathway inhibition and subsequent autophagy activation.	Li et al. [44]
As2O3	Cell line		HL-60	Peripheral blood	Acute promyelocytic leukemia	LC-II	• Hyperoside potentiates both autophagic and apoptotic responses induced by ATO.	Zhang et al. [45]
As2O3	Cell line		HL60	Peripheral blood	Acute promyelocytic leukemia	LC3	• During early treatment, autophagy suppresses ATO-triggered apoptosis, whereas sustained autophagic activation amplifies the apoptotic response.	LIANG, GAO [46]
As2O3	Cell lines		K562	Bone Marrow	Chronic myelogenous leukemia	LC3, p62, Beclin1	• S100A8 functions as a pro-autophagic protein, promoting cell survival and chemoresistance by disrupting the Beclin-1–Bcl-2 interaction. • Silencing S100A8 followed by ATO treatment reduces Beclin-1 and LC3-II levels while elevating p62 expression.	Yang et al. [36]
			HL-60	Peripheral blood	Acute promyelocytic leukemia			
	Childhood AML and ALL bone marrow samples							
Tetraarsenic hexaoxide (As4O6)	Cell line		U937	Pleura/Pleural Effusion, Lymphocyte, Myeloid	Histiocytic lymphoma	Beclin-1, LC-3	• Arsenic tetroxide (As₄O₆) triggers Beclin-1-mediated autophagy along with caspase-dependent apoptotic pathways. • Both autophagy and apoptosis were mitigated by Bcl-2 overexpression or treatment with N-acetylcysteine (NAC).	Han et al. [47]
Sodium arsenite	Cell line		U937	Pleura/Pleural Effusion, Lymphocyte, Myeloid	Histiocytic lymphoma	LC3, p62	• Mitochondria-derived superoxide (O₂°⁻) mediates arsenic-induced autophagy.	Guidarelli et al. [38]
As2O3	Cell line		U937	Pleura/Pleural Effusion, Lymphocyte, Myeloid	Histiocytic lymphoma	LC3	• Reactive oxygen species (ROS) contribute to apoptosis in U937 cells and to autophagy in BM2 cells following ATO exposure. • Arsenic trioxide triggers autophagic cell death in BM2 cell models.	Ondrousková et al. [39]
			V mybtransformed chicken BM2 monoblasts					
As2O3	Cell line		U937	Pleura/Pleural Effusion, Lymphocyte, Myeloid	Histiocytic lymphoma	BNIP3 LC3	• In U937 cells, BNIP3 mRNA levels were elevated by MG132 at EC50, whereas combined treatment with ATO EC50 or MG132 EC25 + ATO EC25 caused a modest reduction. • BNIP3 transcripts were minimally expressed in Raji cells and remained unchanged following treatment with MG132, ATO, or their combination.	Cavaliere et al. [48]
			Raji cells	Lymphoblast	Burkitt's lymphoma			
As2O3	Cell line		Raji cells	Lymphoblast	Burkitt's lymphoma	LC3-Ⅱ, Beclin-1, P62, Hsc70, LAMP-2A	• ATO treatment increased LC3-II and Beclin-1 levels compared to controls, while reducing P62 protein abundance. • Hsc70 and LAMP-2A expression showed minimal changes after ATO exposure.	Li et al. [49]
As2O3	Cell line		Raji cells	Lymphoblast	Burkitt's lymphoma	P62,Beclin1,LC3	• ATO promoted P62 degradation and enhanced Beclin-1 expression.	Li et al. [49]
As2O3 Sodium arsenite (NaAsO2)	Cell line		P3HR1	Ascites	Burkitt's lymphoma	Beclin-1, LC3	• Arsenic trioxide induced autophagic cell death, whereas sodium meta-arsenite (NaAsO₂) activated caspase-dependent apoptosis. • Both ATO and NaAsO₂ increased LC3B expression. • Beclin-1 cleavage occurred in NaAsO₂-treated cells, but remained intact in ATO-treated cells. • Caspase-mediated Beclin-1 cleavage suppresses Beclin-1-dependent autophagy and increases apoptosis susceptibility.	Zebboudj et al. [50]
			Ramos (RA 1) cells	-	Burkitt's lymphoma			
As2O3	Cell lines		K562	Bone Marrow	Chronic myelogenous leukemia	LC3	• Resveratrol potentiates the apoptotic effects of ATO without affecting arsenic-induced autophagic cell death.	Wu et al. [51]
			KT1	Peripheral blood	Chronic myelogenous leukemia			
			U937	Pleura/Pleural Effusion, Lymphocyte, Myeloid	Histiocytic lymphoma			
As2O3	Cell lines		U937		Histiocytic lymphoma	Beclin-1 LC3	• The combined treatment of ATO and sorafenib significantly decreased the viability of U937 and KG-1 cells. • In U937 cells, the expression of selective autophagy genes ULK1 and Beclin1 was reduced, whereas LC3-II levels were elevated.	Haghi et al. [52]
As2O3	Cell lines		K562 and its drug-resistant line K562/ADM cells		Chronic myelogenous leukemia	Beclin-1 LC3	• Bcl-2 potentially contributes to Beclin-1 accumulation and promotes autophagic cell death during ATO treatment.	Cheng et al. [53]
As2O3	Cell lines		U937	Pleura/Pleural Effusion, Lymphocyte, Myeloid	Histiocytic lymphoma	Beclin 1 Atg7	• Blocking autophagy pharmacologically or by targeting Beclin-1 or Atg7 mitigates ATO-induced suppression in AML cell lines and primary leukemic progenitors. • ATO-mediated autophagy requires MEK/ERK activation, while AKT/mTOR or JNK pathways are not essential.	Goussetis et al. [54]
			KT1	Peripheral blood	Chronic myelogenous leukemia			
			MEFs	Embryo Fibroblast	(Immortalized mouse embryonic fibroblasts)			
	Peripheral blood from patients with AML							
As2O3	Cell line	K562 cells and their initiating cells (K562s).		Bone Marrow	Chronic myelogenous leukemia	LC3-B p62	• Low-dose ATO (≤0.5 μM) stimulated acidic vesicular organelle formation in K562s cells, yet inhibited AVO formation in parental K562 cells. • Changes in LC3-B and P62 levels confirmed these differential effects. • The dose of ATO influences whether cells undergo differentiation, malignant transformation, or senescence.	Guo et al. [40]
As2O3	Cell line	The BCR-ABL expressing K562 human leukemia cell line			Chronic myelogenous leukemia	p62, Atg7, LC3	• ATO mediates BCR-ABL degradation through p62/SQSTM1-dependent delivery of the oncoprotein to autolysosomes. • Cathepsin B mediates the oncoprotein degradation of BCR-ABL in this process.	Goussetis et al. [42]
Realgar NPs	Cell line	K562		Bone Marrow	Chronic myelogenous leukemia	LC3,P62	• Realgar nanoparticles reduce BCR-ABL levels through combined induction of apoptosis and autophagy. • Realgar NP-induced autophagy involves modulation of the PI3K/Akt/mTOR signaling pathway.	Shi et al. [55]
As2O3	Cell line	K562		Bone Marrow	Chronic myelogenous leukemia	LC3 P62	• In K562 and MGC803 cells, ATO triggers autophagy via proteasome-dependent degradation of FLIP_L.	Zhang et al. [41]
		Jurkat cells		Peripheral Blood	Acute T cell leukemia			
As2O3	Cell line	Molt-4 cells		-	Acute lymphoblastic leukemia	Beclin-1,	• Bax contributes to Beclin-1 accumulation and the initiation of autophagic cell death in cells treated with ATO.	Qian et al. [56]

### 7.2 Brain cancer

Glioblastoma (GBM) remains highly lethal despite multimodal therapy, with only modest improvements in median survival and persistently low 5-year survival rates. Tumor invasiveness and resistance to apoptosis-driven therapies contribute significantly to poor outcomes. The poor outlook of GBM is partly due to its resistance to apoptosis. **ATO induces cell death characterized by excessive autophagic flux (potentially progressing to autosis) in GBM cells**, a process enhanced by inhibitors of late-stage autophagy, such as chloroquine (CQ). This suggests that trapping cells with an overwhelmed, incomplete autophagy process is deadly. The stage at which autophagy is blocked is crucial—early inhibition offers protection, while late inhibition leads to cell death—highlighting the dynamic nature of the switch. Recent studies demonstrate that arsenic trioxide (ATO) induces **autophagy-associated cell death** in glioma models, often accompanied by upregulation of BNIP3, a pro-death BH3-only protein implicated in arsenite-mediated cytotoxicity [57], [58], [59].

Loss or mutation of key autophagy or tumor-suppressor genes, such as Beclin-1 and PTEN, is common in tumors. In PTEN-deficient, p53 wild-type U87MG cells, arsenite increases ROS, triggers autophagy, and promotes mitochondrial membrane permeabilization, indicating PTEN-independent autophagic mechanisms. Interestingly, arsenite selectively inhibits lysosomal cathepsin B, leading to accumulation of undegraded autophagolysosomal substrates and shifting cells toward apoptosis [60].

Stage-specific modulation of autophagy influences ATO cytotoxicity: early inhibition (e.g., 3-MA or Beclin-1 knockdown) reduces ATO toxicity, whereas blocking late autophagic flux (e.g., chloroquine or Rab7 knockdown) enhances cytotoxicity through vacuole accumulation and apoptosis. Rab7 is essential for late endosomal maturation, and Beclin-1 depletion attenuates both autophagy and apoptosis, abrogating the ATO–chloroquine synergy. Survivin overexpression, linked to glioma progression and poor prognosis, suppresses autophagy and apoptosis and modulates ATO sensitivity [61]. Combined therapies, such as ATO with ionizing radiation or autophagy modulators, enhance mitotic arrest and cell death in resistant gliomas [62]. Sub-cytotoxic cyclosporine A potentiates ATO-induced **autophagy-associated death** in U87MG cells via increased LC3-II and Beclin-1, and silencing DNA repair gene XPC sensitizes U87 cells to UV and ATO, concomitant with elevated autophagy and senescence (Table 2) [59].

**Table 2:** Summary of included studies in brain disorders.

Contaminant	Study model	Cell line			Tissue	Disease	Genes	Findings	Ref
As2O3	Cell lines	U87			Brain	Likely glioblastoma	LC3	• Silencing of the XPC gene increases the susceptibility of U87 cells to autophagic cell death triggered by ATO.	Liu et al. [59]
As2O3	Cell lines	U118-MG			Brain	Glioblastoma	LC3	• Autophagy induced by ATO and ionizing radiation in glioblastoma cells involves activation of the PI3K/Akt and ERK1/2 pathways.	Chiu et al. [62]
As2O3	Cell line	U118-MG			Brain	Glioblastoma	LC3 Atg5 Atg5–12 Beclin1	• Treatment with ATO elevates protein levels of LC3-II, p62, Beclin-1, Atg5, and the Atg5–Atg12 conjugate. • ATO triggers autophagy through suppression of PI3K/Akt signaling and concurrent activation of the MAPK pathway. • Survivin inhibits both autophagic and apoptotic processes in glioblastoma cells.	Chiu et al. [61]
	Mice	U118-MG tumor-model			Brain	Glioblastoma			
As2O3	Cell lines	U118MG			Brain	Glioblastoma	HSP-70	• Arsenic exposure elevates HSP-70 expression during autophagy in U118 glioblastoma cells.	Cheng et al. [63]
As2O3	Cell lines	U87			Brain	Likely glioblastoma	Beclin 1 LC3 SQSTM1	• ATO stimulates autophagy in a concentration-dependent fashion. • Blocking late-stage autophagy, without affecting initial Beclin-1 levels, enhances the cytotoxicity of ATO.	Li et al. [64]
		U251			Brain	Glioblastoma			
As2O3	Cell lines	U87-MG			Brain	Likely glioblastoma	LC3 Lamp-1	• Arsenic-mediated toxicity involves lysosomal cathepsin B inhibition and a dynamic interplay between autophagy and apoptosis.	Pucer et al. [60]
As2O3	Cell lines	DBTRG-05MG			Brain	Glioblastoma	LC3 Atg7	• ROS-dependent autophagy underlies the synergistic cytotoxicity observed with combined ATO and BCNU treatment. • These synergistic effects are abolished by autophagy inhibition, including Atg7 knockdown, 3-MA, or bafilomycin A1 treatment.	Kuo et al. [65]
As2O3	Cell lines	U87-MG			Brain	Likely glioblastoma	-	• Co-administration of ATO with the autophagy inhibitor bafilomycin A1 potentiates its antitumor activity by promoting apoptosis.	Kanzawa et al. [66]
		A172			Brain	Glioblastoma			
		T98G			Brain	Glioblastoma multiforme			
		U373-MG			Brain	Glioblastoma			
		GB-1			Brain	Glioma			
		U251-MG			Brain	Glioblastoma			
As2O3	Cell lines	U373-MG			Brain	Glioblastoma	LC3 BNIP3 BNIP3L	• ATO triggers autophagic cell death in glioma cells via upregulation of the mitochondrial pro-death protein BNIP3.	Kanzawa et al. [67]
		U87-MG			Brain	Likely glioblastoma			
		T98G			Brain	Glioblastoma multiforme			
As2O3	Cell lines	U-373 MG			Brain	Glioblastoma	Atg5 Beclin 1 BNIP3 Atg5-Atg12 LC3 II	• Mitochondrial stress induced by linamarase, linamarin, or glucose oxidase initiates organelle degradation, triggering mitophagy and cell death.	Gargini et al. [68]
		U-87 MG			Brain	Glioblastoma			
	Mice	MCF7lis			Xenograft model	Glioma			
Sodium arsenite	Rats	-			CNS	Neurotoxicity	LC3-II	• Melatonin-mediated autophagy inhibition prevents arsenite-induced reductions in α-synuclein.	Teng et al. [69]
	Primary cultured cortical neurons								
Sodium arsenite	Wistar rats		-	CNS		Neurotoxicity	LC3-II, Atg7, Atg12	• Mild arsenic-induced autophagy at low concentrations enhances spatial learning.	BonakdarYazdi et al. [70]
Sodium arsenite	Primary cultured cortical neurons			CNS		Neurotoxicity	LC3-II, Atg7	• Arsenic-driven autophagy reduces α-synuclein, potentially creating a feedback loop that exacerbates neurotoxicity.	Teng et al. [71]
Sodium arsenite	Cell line		SH-SY5Y cells	CNS		Neurotoxicity	LC3-I/II, P62	• Arsenic exposure simultaneously triggers autophagy initiation and inhibits autophagic flux, leading to accumulation of LC3-I/II and P62. • Blockade of autophagic flux by arsenic promotes the buildup of toxic α-synuclein oligomers, elevating the risk of synucleinopathies.	Cholanians et al. [72]
	Mice		-						

### 7.3 Ovarian cancer

Emerging evidence indicates that arsenic trioxide (ATO) induces dose-dependent autophagic vacuole formation in ovarian carcinoma cells; for instance, treatment with 4 µM ATO for 72 h triggered autophagy in approximately 30–50 % of cells [73], [74], [75]. ATO upregulates autophagy-promoting factors, including Beclin-1 and ATG5, in a time- and dose-dependent manner. Concurrently, it downregulates oncogenic or signaling molecules such as EVI1, TAK1, SMAD2/3, and TGFβRII, while increasing SnoN/SkiL levels. Notably, SnoN expression temporally parallels LC3-II accumulation, appearing early during cytoplasmic vacuolation prior to apoptosis. Functional assays show that Beclin-1 knockdown minimally affects autophagy, whereas silencing ATG5, ATG7, or hVps34 substantially suppresses ATO-induced autophagy. SnoN depletion reduces LC3-II and enhances PARP cleavage, suggesting a Beclin-1-independent autophagic pathway in ovarian carcinoma, with SnoN maintaining an autophagy-dependent survival program [73].

Given the central role of the PI3K/AKT/mTOR axis in ovarian tumorigenesis, mTOR inhibitors such as everolimus (Rad001) have limited efficacy as monotherapies. Remarkably, combining ATO with Rad001 produces synergistic cytotoxicity, associated with decreased p-AKT and increased autophagy markers (ATG5–ATG12 conjugate, LC3-II), highlighting a strategy to sensitize tumors to targeted therapy [76]. PENAO, an organoarsenical in phase-1 evaluation, exhibits variable activity across ovarian cancer histotypes; SKOV-3 cells demonstrate resistance due to PENAO-induced oxidative stress, upregulation of heme oxygenase-1, and a glycolytic metabolic shift. Co-treatment with an mTORC1 inhibitor reverses this adaptive response, restoring sensitivity and promoting cell death via combined autophagic and apoptotic mechanisms. These findings support dual-targeting of mitochondria and mTOR pathways in refractory epithelial ovarian cancers (Table 3) [77]

**Table 3:** Summary of included studies in ovarian cancers.

Contaminant	Study model	Cell line	Tissue		Disease	Marker	Findings	Ref
As2O3	Cell line	SKOV-3	Ovary		Ovarian carcinoma	ATG5–ATG12 conjugate, LC3–2,p62	• Combined treatment with ATO and everolimus (Rad001) enhances cytotoxicity in ovarian cancer cells by simultaneously promoting autophagy and apoptosis.	Liu et al. [76]
	Mice	Ovarian cancer xenograft						
PENAO		SKOV-3		Ovary	Ovarian carcinoma	LC3	• A dual-targeting therapeutic approach aimed at both mitochondria and mTOR—using an mTORC1 inhibitor together with PENAO—shows promise for treating recurrent or drug-resistant epithelial ovarian cancers. • The combination of these two agents acts synergistically to promote cell death through coordinated activation of autophagic and apoptotic pathways.	Decollogne et al. [77]
		OVCAR-3		Ovary	Ovarian carcinoma			
		CH-1		Ovary	Ovarian carcinoma			
		EFO27		Ovary	Ovarian mucinous adenocarcinoma			
	Mice	-		Ovarian cancer xenograft				
As2O3	Cell lines	SKOV-3		Ovary	Ovarian carcinoma	LC3, Beclin-1, ATG5, ATG7, hVps34, p62	• ATO activates a Beclin-1–independent autophagic mechanism in which SnoN facilitates autophagy-mediated cell survival. Silencing of ATG7, ATG5, or hVps34 markedly suppresses ATO-induced autophagy.	Smith et al. [73]
		HEY		Ovary	Papillary cystadenocarcinoma of the ovary			
		OVCA429		Ovary	Ovarian cystadenocarcinoma			
As2O3	Cell line	CHO AA8		Ovary	-	-	• Arsenic trioxide triggers both autophagic activity and mitotic cell death.	Izdebska et al. [78]
Arsenite	Cell line	CHO		Ovary	-	p62 LC3	• Cyclic AMP phosphodiesterase-4A4 (PDE4A4) and p62 are recruited to a reversible protein aggregate associated with both proteasomal degradation and autophagic processing.	Christian et al. [79]

### 7.4 Prostate cancer

Arsenic trioxide (ATO) activates autophagic pathways in prostate carcinoma models [80]. Although mTOR inhibitors such as everolimus (Rad001) exhibit limited efficacy as single agents, co-treatment with ATO enhances cytotoxicity through concurrent activation of autophagy and apoptosis. This synergistic effect is associated with increased Beclin-1 mRNA stability and upregulation of autophagy effectors, including the ATG5–ATG12 conjugate and LC3-II. In vivo, the Rad001–ATO combination effectively suppresses tumor growth in prostate cancer xenografts, providing mechanistic rationale for combining arsenic derivatives with PI3K/AKT/mTOR pathway inhibitors [74].

Separately, silibinin modulates oxidative balance in arsenic-exposed human prostate cancer (PCa) cells, inhibiting proliferation and survival via induction of both autophagy and apoptosis. Silibinin downregulates cyclin-dependent kinases (CDK2, CDK4, CDK6) and cyclins (D1, D3, E), leading to G1 or G2/M cell cycle arrest and concurrent upregulation of CDK inhibitors p21 and p27. KML001 (sodium meta-arsenite, NaAsO₂), an orally bioavailable arsenic compound, triggers dose- and time-dependent apoptosis and autophagy in prostate cancer cells; co-treatment with the ROS scavenger N-acetylcysteine (NAC) attenuates LC3 accumulation and PARP cleavage, highlighting an ROS-mediated mechanism (Table 4) [81].

**Table 4:** Summary of included studies in prostate carcinoma.

Contaminant	Study model	Cell line	Tissue	Disease	Marker	Findings	Ref
As2O3	Cell lines	LNCaP	Prostate	Prostate carcinoma	ATG5-ATG12 conjugate, Beclin1, LC3	• The combined administration of arsenic trioxide and Rad001 produces a synergistic enhancement of both autophagic and apoptotic pathways.	Tai et al. [74]
		PC3	Prostate	Prostate carcinoma			
	Mice		LNCaP xenograft model.				
As2O3	Cell line	PC3	Prostate	Adenocarcinoma	Atg5, Beclin 1, BNIp3, Atg5-Atg12, LC3–2	• Cellular exposure to linamarase, linamarin, or glucose oxidase induces mitochondrial injury, initiating organelle degradation and activating mitophagy that culminates in cell death.	Gargini et al. [68]
Sodium arsenite	Cell lines	DU145	Prostate	Carcinoma	Beclin 1	• Treatment with silibinin or arsenic—either individually or in combination—upregulates Beclin-1 expression and initiates autophagy-dependent cell death.	Prajapati et al. [81]
		22Rv1	Prostate	Carcinoma			
KML001 (NaAsO2, sodium metaarsenite,kominox)	Cell lines	PC3	Prostate	Prostate carcinoma	LC3	• KML001 triggers autophagic activity through activation of oxidative stress–mediated signaling cascades.	You et al. [82]
		DU145	Prostate	Prostate carcinoma			
		LNCaP	Prostate	Prostate carcinoma			
	Mice	-	DU145 xenograft model				

### 7.5 Skin cancer

Epidemiological and mechanistic evidence links arsenic exposure to skin carcinogenesis, although precise molecular pathways remain incompletely defined. In keratinocytes, arsenic trioxide (ATO) combined with BCNU (carmustine) enhances cytotoxicity via **autophagy-associated cell death**, and antioxidant treatment abrogates both ROS generation and autophagy, highlighting a redox-dependent mechanism [65]. The scaffold protein p62/SQSTM1 integrates signals from mTOR, MAPK, and NF-κB and plays a central role in Nrf2-mediated antioxidant responses. In HaCaT cells, arsenic induces p62 expression independently of autophagy, and chronic exposure in mice elevates epidermal p62 alongside increased proliferation. p62 knockdown reduces arsenic-driven Nrf2 activation and induces sustained p21 upregulation, modestly affecting apoptosis while significantly inhibiting proliferation, suggesting p62 as a potential target to prevent arsenic-induced skin tumorigenesis [83]. In this scenario, inhibiting the autophagy switch or fixing its impairment could serve as a promising chemopreventive strategy.

Comparative studies of realgar nanoparticles and ATO in melanoma cell lines (BOWES, A375) demonstrate dose-dependent cytotoxicity: lower doses enhance lysosomal activity and autophagy, whereas higher doses induce apoptosis. Realgar also modulates cell cycle progression, causing G2/M arrest, and alters phosphorylation of key signaling kinases (IκB, Akt, ERK1/2, p38, JNK), indicating perturbation of multiple pathways (Table 5) [84], [85], [86].

**Table 5:** Summary of included studies in skin cancer.

Contaminant	Study model	Cell line	Tissue	Disease	Marker	Findings	Ref
Sodium arsenic	Cell line	HaCaT	Immortalized human keratinocytes	-	p62, LC3	• Arsenic exposure elevates p62 expression, thereby modulating the Nrf2 signaling cascade. Therapeutic targeting of p62 could represent a potential strategy to mitigate or prevent arsenic-induced cutaneous carcinogenesis.	Shah et al. [83]
	Mice	-	Skin	-			
As2O3	Cell lines	A2058	Skin	Melanoma	LC3 Atg7	• Reactive oxygen species–dependent autophagic signaling mediates the synergistic cytotoxic effects of arsenic trioxide and BCNU. Inhibition of autophagy using Atg7-specific siRNA or pharmacologic blockers such as 3-MA and bafilomycin A1 partially abrogates ATO + BCNU–induced cell death.	Kuo et al. [65]
Realgar Nps, As2O3	Cell lines	BOWES	Skin	Melanoma	LC3-II, p62	• Arsenic exposure at lower doses predominantly triggers autophagic responses, whereas increasing concentrations shift the cellular fate toward apoptotic death, indicating a dose-dependent transition between survival and cytotoxic pathways.	Pastorek et al. [84]
		A375	Skin	Malignant melanoma			

### 7.6 Other cancers

Over the past decade, accumulating genetic and mechanistic evidence has strengthened the link between autophagy dysregulation and tumorigenesis across diverse cancer types. Autophagy-promoting molecules are often associated with tumor-suppressive functions, whereas autophagy inhibitors frequently correlate with oncogenic processes. These findings indicate that autophagy contributes to multiple stages of cancer initiation, progression, and adaptation [86]. Given that tumor cells exploit autophagy to survive metabolic and environmental stresses, defining its precise roles is essential for translating mechanistic insights into effective therapeutic strategies. A comprehensive summary of studies examining autophagy in various cancer models is provided in Table 6.

**Table 6:** Summary of included studies in other cancers.

Contaminant	Study model	Cell line	Tissue		Disease	Marker	Findings	Ref
As2O3	Cell line	HT1080	Connective tissue		Fibrosarcoma	LC3-I, LC3-II, p62, Beclin 1	• Combined treatment with ionizing radiation (IR) and arsenic trioxide (ATO) enhances both autophagy and apoptosis via ERK1/2 activation and concurrent suppression of Akt signaling pathways.	Chiu et al. [87]
	Mice	HT1080 cells xenograft model						
Arsenic sulfide (As2S2)	Cell lines	143B	Bone		Osteosarcoma	GFP-LC3 Beclin-1	• Arsenic sulfide (As₂S₂) elevates Beclin-1 and LC3B-II expression in 143B and MG-63 osteosarcoma cells. The compound activates the ROS/JNK axis while inhibiting Akt/mTOR signaling, thereby initiating a potentially pro-survival autophagic response.	Wang et al. [88]
		MG-63	Bone		Osteosarcoma			
		HOS	Bone		Osteosarcoma			
		U2OS	Bone		Osteosarcoma			
	Mice	-	Osteosarcoma xenograft model					
As2O3	Cell lines	HOS	Bone		Osteosarcoma	Beclin 1, LC3, p62	• The synergistic administration of IR and ATO promotes autophagy and apoptosis by downregulating the PI3K/Akt pathway, highlighting a cooperative mechanism in tumor cell cytotoxicity.	Chiu et al. [89]
As2O3	Cell line	SW 1353	Bone		Chondrosarcoma	LC3-I, LC3-II, p62	• ATO exerts therapeutic effects by inducing G₂/M phase arrest and activating both apoptotic and autophagic pathways mediated through Gli signaling modulation.	Jiao et al. [90]
As2O3	Cell line	Neonatal rat ventricular cardiomyocytes			Cardiomyoblastoma	LC3 p62	• Inhibition of Rho-associated kinase (ROCK) enhances cytoprotective autophagy in cells subjected to arsenic exposure, suggesting a compensatory survival mechanism.	Bessho et al. [91]
Arsenite	Cell lines	HeLa	Cervix		Adenocarcinoma	p62 LC3	• Cyclic AMP phosphodiesterase-4A4 (PDE4A4) and p62 co-localize within a novel, reversible protein aggregate associated with both proteasomal degradation and autophagic processing pathways.	Christian et al. [79]
As2O3	Cell lines	HT-29		Colorectal adenocarcinoma		LC3 Atg7	• Reactive oxygen species–dependent autophagic signaling mediates the synergistic cytotoxicity between As₂O₃ and BCNU. Pharmacologic or genetic inhibition of autophagy—using Atg7 siRNA, 3-MA, or bafilomycin A1—partially rescues cells from ATO+BCNU–induced death.	Kuo et al. [65]
	
		MCF-7	Mammary gland /Breast		Adenocarcinoma			
As2O3	Cell lines	MDA-MB	Mammary gland/Breast		Breast carcinoma	Atg5 Beclin 1 BNIp3 Atg5-Atg12 LC3 II	• Mitochondrial damage induced by the linamarase/linamarin/glucose oxidase system initiates extensive organelle degradation, leading to mitophagy activation and subsequent cell death.	Gargini et al. [68], [86]
		HeLA	Cervix		Adenocarcinoma			
		MCF7	Mammary gland /Breast		Adenocarcinoma			
	Mice	MCF7lis	Xenograft Model		Breast cancer			
As2O3	Cell line	MCF7	Breast		Breast adenocarcinoma	-	• Arsenic trioxide treatment effectively reverses fibroblast-mediated tamoxifen resistance, restoring drug sensitivity in resistant tumor cell populations.	Martinez-Outschoorn et al. [86]

## 8 Therapeutic implications and future perspectives

The Autophagy Switch model has direct translational relevance. The goal is not to universally inhibit or promote autophagy, but to strategically modulate it based on context (Fig. 3). As seen with ATO and everolimus, targeting parallel pathways can trigger lethal autophagy in resistant cancers [74]. The opposing effects of early vs. late-stage autophagy inhibitors in GBM models underscore the need for precision. Biomarkers of autophagic flux are essential to determine the optimal therapeutic window. Tumors may resist ATO by upregulating protective autophagy. In such cases, combining ATO with autophagy inhibitors, such as hydroxychloroquine, could be beneficial. Conversely, in tumors where ATO fails to induce sufficient autophagy, sensitizing agents may be required [92], [93].

## 9 Future research directions

Future research and efforts may focus on developing arsenic-specific autophagy flux reporters for real-time in vivo tracking, defining the Switch thresholds that systematically determine the genetic and metabolic conditions guiding the survival-death decision, and exploring novel arsenic formulations such as nano-particle realgar and organoarsenicals like PENAO that may engage the autophagy switch differently than ATO, offering new therapeutic possibilities.

## 10 Discussion

This review integrates current evidence linking arsenic exposure and treatment to autophagy modulation across diverse disease contexts. Arsenic is a potent inducer of oxidative stress, driving DNA damage, lipid peroxidation, and apoptosis. While arsenic-induced apoptotic mechanisms are well characterized, the contribution of autophagy to arsenic toxicity or therapeutic efficacy has been less thoroughly explored. Impaired autophagy can cause cellular dysfunction and increase cancer susceptibility, whereas some anticancer interventions exploit autophagy as a cell-death pathway complementary to apoptosis [94].

Apoptosis and autophagy intersect at multiple nodes, sharing key effector proteins such as Bcl-XL, Bcl-2, ATG5, Beclin-1, and BNIP3, and converging on upstream signaling pathways including PI3K/Akt/mTOR, MAPK, STAT3, and NF-κB [89], [95]. Arsenic trioxide often suppresses Akt/mTOR activity, thereby relieving autophagic inhibition. Oxidative stress modulates regulators such as PTEN, p70S6K, and ERK1/2, contributing to autophagy induction. ROS target nucleic acids, proteins, and lipids, activating both apoptotic and autophagic programs via ROS–JNK signaling with concurrent Akt/mTOR suppression. In renal proximal tubular HK-2 cells, autophagy acts as an early adaptive response to arsenic-induced oxidative stress, subsiding once the oxidative burden is alleviated [89], [96], [97], [98].

The transcription factor Nrf2 plays a context-dependent role: while generally cytoprotective, prolonged As³ ⁺ exposure can shift Nrf2 signaling toward tumor-promoting activity. Accumulation of p62 due to impaired autophagy further enhances Nrf2 activation, favoring cell survival, proliferation, and carcinogenesis in transformed cells [99]. Collectively, these findings highlight autophagy’s involvement in diverse human cancers and disorders, emphasizing that therapeutic modulation of autophagy—whether stimulation or inhibition—must be carefully tailored to disease stage, tissue type, and timing of arsenic exposure or treatment.

## 11 Conclusion

Elucidating whether autophagy modulation meaningfully contributes to tumor suppression or oncogenesis in the context of arsenic exposure is of critical importance. As a key adaptive mechanism, autophagy enables tumor cells to withstand metabolic and environmental stress, underscoring the need for a nuanced understanding of autophagic dynamics to advance cancer research and inform therapeutic strategies [100]. Insights from the literature summarized in this review may guide clinicians in determining when autophagy activators or inhibitors could confer therapeutic benefit in arsenic-related pathologies. Moving forward, precise quantification of autophagic flux in clinically relevant models, alongside careful translation of experimental findings to human studies, will be essential for improving patient outcomes.

## 12 Abbreviations

Abbreviations used in this article include: Ambra1 (activating molecule in Beclin-1–regulated autophagy), Atg (autophagy-related genes), Bcl (B-cell leukemia/lymphoma), FIP200 (focal adhesion kinase family–interacting protein of approximately 200 kDa), LC3 (microtubule-associated protein 1 light chain 3), mTOR (mammalian or mechanistic target of rapamycin), PI3K (phosphatidylinositol 3-kinase), ULK1 (Unc-51-like autophagy activating kinase 1), and Vps (vacuolar protein sorting). Other abbreviations include Akt1 (thymoma viral proto-oncogene 1), Bad (BCL2-associated agonist of cell death), Bak1 (BCL2-antagonist/killer 1), Bax (BCL2-associated X protein), Bcl2 (B-cell leukemia/lymphoma 2), Bcl2l1 (BCL2-like 1), Becn1 (Beclin 1, autophagy-related), and Bid (BH3-interacting domain death agonist). Caspase family members such as Casp3 (caspase 3) and Casp8 (caspase 8) are also mentioned, along with Cdkn1b (cyclin-dependent kinase inhibitor 1B), Cdkn2a (cyclin-dependent kinase inhibitor 2 A), Cln3 (ceroid lipofuscinosis neuronal 3, juvenile form—Batten or Spielmeyer–Vogt disease), Gabarap (gamma-aminobutyric acid receptor–associated protein), Igf1 (insulin-like growth factor 1), Ins2 (insulin II), Mtor (mechanistic target of rapamycin, serine/threonine kinase), Nfkb1 (nuclear factor of kappa-light polypeptide gene enhancer in B-cells 1), Pten (phosphatase and tensin homolog), Rab24 (member of the RAB family, RAS oncogene–related protein 24), Rb1 (retinoblastoma 1), Sqstm1 (sequestosome 1), Tgfb1 (transforming growth factor beta 1), Tnf (tumor necrosis factor), and Trp53 (transformation-related protein 53, p53).

## CRediT authorship contribution statement

**Hamed Zeinvand-Lorestani:** Writing – review & editing. **Fakher Rahim:** Writing – review & editing. **Marzieh Zeinvand-Lorestani:** Writing – review & editing, Writing – original draft, Data curation.

## Declaration of Competing Interest

The authors declare that they have no known competing financial interests or personal relationships that could have appeared to influence the work reported in this paper.

## Data Availability

Data will be made available on request.
